# The association of hyperglycaemia and insulin resistance with incident depressive symptoms over 4 years of follow-up: The Maastricht Study

**DOI:** 10.1007/s00125-020-05247-9

**Published:** 2020-08-05

**Authors:** Anouk F. J. Geraets, Sebastian Köhler, Rutendo Muzambi, Casper G. Schalkwijk, Anke Oenema, Simone J. P. M. Eussen, Pieter C. Dagnelie, Coen D. A. Stehouwer, Nicolaas C. Schaper, Ronald M. A. Henry, Carla J. H. van der Kallen, Anke Wesselius, Annemarie Koster, Frans R. J. Verhey, Miranda T. Schram

**Affiliations:** 1 Department of Psychiatry and Neuropsychology, Maastricht University Medical Center (MUMC+), Maastricht, the Netherlands; 2 Department of Internal Medicine, Maastricht University Medical Center (MUMC+), Maastricht, the Netherlands; 3 School of Mental Health and Neuroscience (MHeNs), Faculty of Health, Medicine & Life Sciences, Maastricht University, Maastricht, the Netherlands; 4 School for Cardiovascular Diseases (CARIM), Faculty of Health, Medicine & Life Sciences, Maastricht University, Maastricht, the Netherlands; 5 Faculty of Epidemiology & Population Health, London School of Hygiene & Tropical Medicine, London, UK; 6 Department of Health Promotion, Maastricht University Medical Center (MUMC+), Maastricht, the Netherlands; 7 Care and Public Health Research Institute (CAPHRI), Faculty of Health, Medicine & Life Sciences, Maastricht University, Maastricht, the Netherlands; 8 School of Nutrition and Translational Research in Metabolism (NUTRIM), Faculty of Health, Medicine & Life Sciences, Maastricht University, Maastricht, the Netherlands; 9 Department of Epidemiology, Maastricht University Medical Center (MUMC+), Maastricht, the Netherlands; 10 Heart and Vascular Center, Maastricht University Medical Center (MUMC+), Maastricht, the Netherlands; 11 Department of Genetics & Cell Biology, Complex Genetics, Maastricht University Medical Center (MUMC+), Maastricht, the Netherlands; 12 Department of Social Medicine, Maastricht University Medical Center (MUMC+), Maastricht, the Netherlands

**Keywords:** Depression, Depressive symptoms, Epidemiology, Hyperglycaemia, Insulin resistance, Population-based cohort study, Type 2 diabetes mellitus

## Abstract

**Aims/hypothesis:**

Depression is twice as common in individuals with type 2 diabetes as in the general population. However, it remains unclear whether hyperglycaemia and insulin resistance are directly involved in the aetiology of depression. Therefore, we investigated the association of markers of hyperglycaemia and insulin resistance, measured as continuous variables, with incident depressive symptoms over 4 years of follow-up.

**Methods:**

We used data from the longitudinal population-based Maastricht Study (*n* = 2848; mean age 59.9 ± 8.1 years, 48.8% women, 265 incident depression cases, 10,932 person-years of follow-up). We assessed hyperglycaemia by fasting and 2 h post-load OGTT glucose levels, HbA_1c_ and skin autofluorescence (reflecting AGEs) at baseline. We used the Matsuda insulin sensitivity index and HOMA-IR to calculate insulin resistance at baseline. Depressive symptoms (nine-item Patient Health Questionnaire score ≥10) were assessed at baseline and annually over 4 years. We used Cox regression analyses, and adjusted for demographic, cardiovascular and lifestyle risk factors.

**Results:**

Fasting plasma glucose, 2 h post-load glucose and HbA_1c_ levels were associated with an increased risk for incident depressive symptoms after full adjustment (HR 1.20 [95% CI 1.08, 1.33]; HR 1.25 [1.08, 1.44]; and HR 1.22 [1.09, 1.37] per SD, respectively), while skin autofluorescence, insulin sensitivity index and HOMA-IR were not (HR 0.99 [0.86, 1.13]; HR 1.02 [0.85, 1.25]; and HR 0.93 [0.81, 1.08], per SD, respectively).

**Conclusions/interpretation:**

The observed temporal association between hyperglycaemia and incident depressive symptoms in this study supports the presence of a mechanistic link between hyperglycaemia and the development of depressive symptoms.

**Figure:**
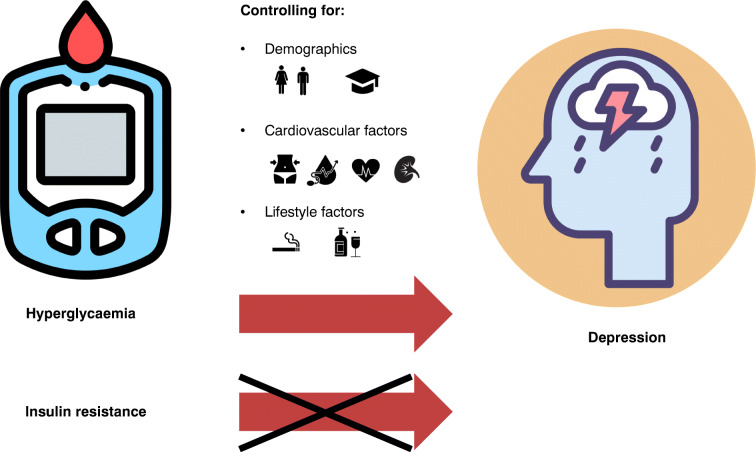
Graphical abstract

**Electronic supplementary material:**

The online version of this article (10.1007/s00125-020-05247-9) contains peer-reviewed but unedited supplementary material, which is available to authorised users.

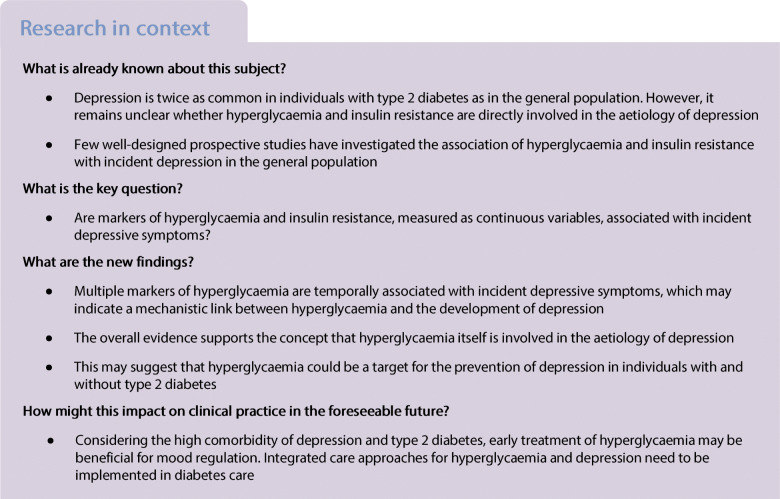



## Introduction

The prevalence of depression is nearly doubled in individuals with type 2 diabetes as compared with the general population, with prevalence rates of 6.5% to 33% [1]. Comorbid depression in type 2 diabetes is associated with impaired quality of life [2], worse self-care, suboptimal blood glucose levels and an increased risk for macro- and microvascular complications, mortality [3] and dementia [4]. In addition, their co-occurrence has an adverse economic impact with increased healthcare costs and decreased work productivity [5]. Furthermore, depression appears to be highly persistent and/or recurrent in type 2 diabetes [6]. Although there is evidence for a bidirectional association between type 2 diabetes and depression, the exact nature and the aetiological direction of the relationship remain unknown [1].

Hyperglycaemia and insulin resistance are key features of type 2 diabetes, and have been proposed as underlying mechanisms involved in the aetiology of depression [7]. Both fluctuations in plasma glucose and prolonged hyperglycaemia may be involved in the development of depression. The brain is particularly vulnerable to fluctuations in plasma glucose levels because neurons do not possess an active glucose transporter. As a consequence, high extracellular glucose levels lead to high intracellular glucose levels. The resulting biochemical changes, for instance the formation of reactive oxygen species (ROS) or AGEs, and accumulation of the resulting damage over the years, may lead to neuronal damage and/or disturbances of the hypothalamic–pituitary–adrenal axis, which eventually may lead to depression [7]. However, current evidence on the temporality of these associations remains scarce. A recent meta-analysis of prospective studies found an association between prevalent diabetes and incident depression but not between impaired glucose metabolism (IGM) or newly diagnosed type 2 diabetes and incident depression, compared with normal glucose metabolism (NGM) [8]. However, numbers for incident depression with IGM [9–11] or newly diagnosed type 2 diabetes were relatively small [10–13] and thus confidence intervals were large, and all studies used categorical instead of continuous values of glucose metabolism.

With regard to insulin resistance, only four prospective studies examined the association with incident depression. One study found an association [14], while the others did not [15–17]. However, these studies have important methodological limitations, such as a single follow-up assessment of depression [14, 16, 17], inclusion of only men [15] or only elderly men [14], a small study population [17] or a small number of incident depression cases [14].

In summary, there is a need for methodologically well-conducted prospective studies to assess whether hyperglycaemia and insulin resistance are temporally related to the development of depression. Therefore, the aim of this study was to examine the associations of markers of hyperglycaemia and insulin resistance measured as continuous variables with incident clinically relevant depressive symptoms within the population-based Maastricht Study. In addition, we assessed whether these associations were independent of demographic, cardiovascular and lifestyle risk factors, or differed between women and men. We hypothesised that hyperglycaemia and higher levels of insulin resistance are independently associated with incident clinically relevant depressive symptoms, and that these associations are similar in women and men.

## Methods

### Study population and design

**Fig. 1:**
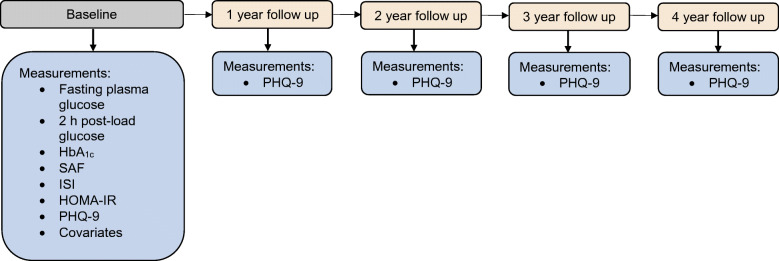
Study design

The Maastricht Study is an observational population-based cohort study. The rationale and methodology have been described previously [18]. In brief, the study focuses on the aetiology, pathophysiology, complications and comorbidities of type 2 diabetes and is characterised by an extensive phenotyping approach. Eligible for participation were all individuals aged between 40 and 75 years and living in the southern part of the Netherlands. Participants were recruited through mass media campaigns, the municipal registries and the regional Diabetes Patient Registry via mailings. Recruitment was stratified according to known type 2 diabetes status, with an oversampling of individuals with type 2 diabetes, for reasons of efficiency. The present report includes baseline data from 3124 participants, who completed the baseline survey between November 2010 and September 2013. Figure 1 gives an overview of the study design. The baseline examinations of each participant were performed within a time window of 3 months. Follow-up data were only available for depression data and were available in 91.9%, 85.4%, 79.9% and 71.4% of the participants with available baseline data at, respectively, 1, 2, 3 and 4 years of follow-up. The study has been approved by the institutional medical ethical committee (NL31329.068.10) and the Minister of Health, Welfare, and Sports of the Netherlands (Permit 131088-105234-PG). All participants gave written informed consent.

**Fig. 2:**
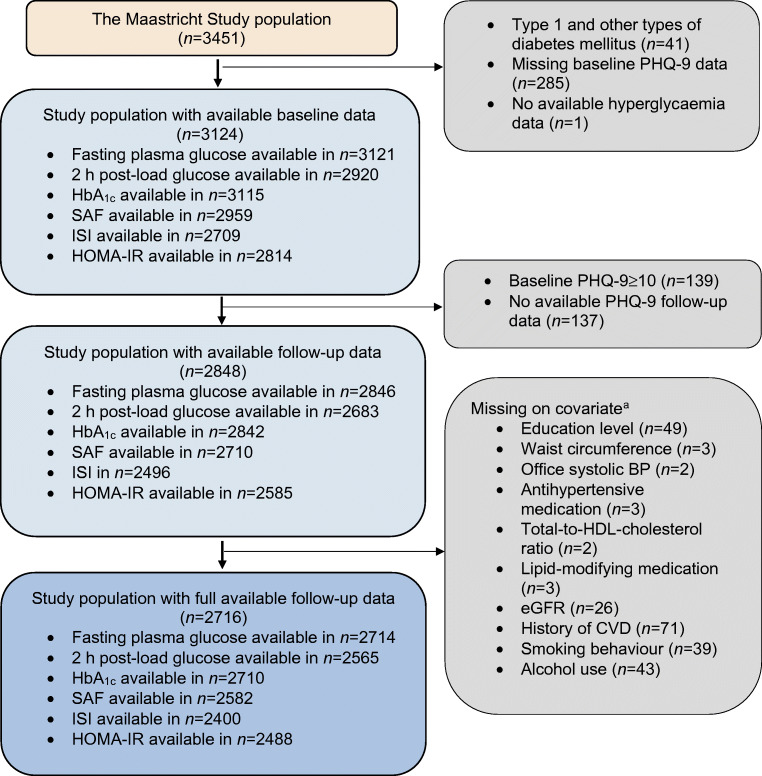
Flowchart of study population. ^a^Missing data on covariates are not mutually exclusive

Figure 2 shows the flowchart of the study population. From the initial 3451 participants we excluded individuals with other types of diabetes than type 2 diabetes (*n* = 41). For the cross-sectional analyses we included participants with available hyperglycaemia, insulin resistance and nine-item Patient Health Questionnaire (PHQ-9) data at baseline (*n* = 3124). For the longitudinal analyses, we excluded participants with clinically relevant depressive symptoms at baseline (PHQ-9 score ≥10, *n* = 139) or without any follow-up PHQ-9 data (*n* = 137) to investigate the associations with newly developed depressive symptoms during follow-up, resulting in a study population of 2848 participants with an average follow-up duration of 3.8 ± 1.0 years.

### Hyperglycaemia

Markers of hyperglycaemia were measured at baseline. Participants, except those who used insulin (as endogenous insulin production is limited), underwent a standardised 2 h 75 g OGTT to determine fasting and 2 h post-load blood glucose levels after an overnight fast. For safety reasons, participants with a fasting glucose level above 11.0 mmol/l, as determined by a finger prick, did not undergo the OGTT (*n* = 42). Venous fasting and 2 h post-load plasma glucose levels were measured by the enzymatic hexokinase method on two automatic analysers, the Beckman Synchron LX20 (Beckman Coulter, CA, USA) for samples obtained between November 2010 and April 2012, and the Roche Cobas 6000 (Roche Diagnostics, Mannheim, Germany) for samples obtained thereafter. Glucose metabolism status was defined according to the World Health Organization 2006 criteria as NGM, prediabetes (fasting glucose 6.1–7.0 mmol/l or 2 h post-load blood glucose 7.8–11.1 mmol/l) or type 2 diabetes (fasting blood glucose ≥7.0 mmol/l or 2 h post-load blood glucose ≥11.1 mmol/l, or used oral glucose-lowering medication or insulin) [19]. Type 1 diabetes and other types of diabetes were determined by use of a clinical interview. HbA_1c_ was determined in fasting venous blood samples by ion-exchange high performance liquid chromatography [18]. Skin autofluorescence (SAF) was measured with the AGE Reader (DiagnOptics Technologies, Groningen, the Netherlands), which is a desktop device that uses ultraviolet light to excite autofluorescence in human skin tissue to estimate the level of AGE accumulation in the skin, as described elsewhere [20].

### Insulin resistance

Insulin resistance was assessed by the Matsuda insulin sensitivity index (ISI) and the HOMA-IR [21] at baseline only. The ISI was calculated as suggested by DeFronzo and Matsuda [22]: ISI = 10,000/(*G*_0_ × *I*_0_ × *G*_mean_ × *I*_mean_)1/2, where *G* and *I* represent plasma glucose (mmol dl^−1^) and insulin (mU l^−1^) concentrations, respectively, and ‘0’ and ‘mean’ indicate fasting value and mean value during OGTT, respectively. The reciprocal (i.e. 1/ISI) was used to reflect insulin resistance as a risk factor. The ISI is strongly correlated (*r* = 0.73, *p* < 0.0001) with the rate of whole-body glucose disposal during the euglycaemic insulin clamp [23].

HOMA-IR was calculated with the HOMA2 calculator version 2.2.3 for Windows [24]. HOMA-IR is the most widely used and validated surrogate marker of insulin resistance and corresponds reasonably well to clamp-derived measures of insulin sensitivity [25]. Neither measure was calculated for participants receiving insulin treatment (*n* = 169); as endogenous insulin levels will be close to zero, ISI and HOMA-IR calculations will result in zero as well.

### Depressive symptoms

Depressive symptoms were assessed by a validated Dutch version of the PHQ-9 [26] both at baseline and during annual follow-up over 4 years. The PHQ-9 is a self-administered questionnaire that assesses the presence of the nine symptoms for the Diagnostic and Statistical Manual of Mental Disorders (DSM-IV) criteria for a major depressive disorder (MDD) [27] on a four-point Likert-scale ranging from 0, ‘not at all’, to 4, ‘nearly every day’. When one or two items were missing, the total score was calculated as 9 × (total points/9 − number of missing items) and rounded to the nearest integer. When more items were missing, the total score was scored as missing.

A cut-off score of ≥10 is most often used as a dichotomous scoring system for defining clinically relevant depressive symptoms, with sensitivity and specificity of, respectively, 88% and 78% [28]. Online PHQ-9 questionnaires were completed annually during a follow-up period of 4 years. Prevalent depressive symptoms were defined as clinically relevant depressive symptoms at baseline (PHQ-9 ≥10). Incident depressive symptoms were defined as no depressive symptoms at baseline (PHQ-9 <10) and presence of clinically relevant depressive symptoms on at least one follow-up moment (PHQ-9 ≥10). In addition, at baseline only, current and lifetime diagnosis of MDD was assessed by the Mini-International Neuropsychiatric Interview (MINI) [29].

### General characteristics and covariates

General characteristics and covariates were measured at baseline. Educational level (low, intermediate, high), partner status (partner/no partner), history of CVD, smoking status (never, current, former), alcohol consumption (none, low, high), physical activity and Mediterranean diet score were assessed by questionnaires [18]. We measured height, weight, waist circumference, office blood pressure, plasma lipid profile, eGFR (in ml min^−1^ 1.73 m^−2^) and 24 h urinary albumin excretion (twice). Urinary albumin excretion was defined as normal (<15 mg/24 h), microalbuminuria (15 to <30 mg/24 h) or macroalbuminuria (≥30 mg/24 h). Medication use was assessed in a medication interview where generic name, dose and frequency were registered. More details about these general characteristics and covariates are provided in the electronic supplementary material (ESM) methods.

### Statistical analysis

All statistical analyses were performed by use of the Statistical Package for Social Sciences (version 25.0; IBM, Chicago, Illinois, USA). General characteristics of the study population were evaluated using independent *t* tests, Mann–Whitney *U* tests or *χ*^2^ tests. Negative binomial and logistic regression analyses were used to investigate the cross-sectional associations of markers of hyperglycaemia and insulin resistance per SD with, respectively, depressive symptoms and clinically relevant depressive symptoms. We used Cox proportional regression analyses to assess the association of markers of hyperglycaemia and insulin resistance per SD with incident depressive symptoms (PHQ-9 ≥10), with time-in-study as time axis. Participants were censored at the date of the event or, in case of attrition, the last available date of follow-up, whichever came first. Hazard ratios indicate the increased risk for incident depressive symptoms per SD higher marker of hyperglycaemia or insulin resistance. We performed complete case analyses in which associations were adjusted for potential confounders in four models: model 1, crude; model 2, adjusted for demographic confounders (age, sex and educational level); model 3, additionally adjusted for cardiovascular risk factors (waist circumference, office systolic blood pressure, blood pressure-lowering medication, total-to-HDL-cholesterol ratio, lipid-modifying medication, eGFR and history of CVD); and model 4, additionally adjusted for modifiable lifestyle-related risk factors (smoking behaviour and alcohol use). We also investigated whether there was an interaction with sex in the fully adjusted model.

Several additional analyses were performed. To study whether the associations were driven by the oversampling of individuals with diagnosed type 2 diabetes, we additionally adjusted for type 2 diabetes, and excluded participants with type 2 diabetes from the analyses. To reduce potential misclassification of participants with subthreshold depression (MDD but low PHQ-9 scores due to remission or treatment), we performed the following sensitivity analyses; first, we additionally adjusted for use of antidepressant medication at baseline; second, we excluded participants who used antidepressant medication at baseline; and third, we excluded participants who had an MDD diagnosis at baseline. To restrict analyses to ‘de novo’ depression, we excluded participants who had a lifetime MDD diagnosis. We also applied stricter rules on the follow-up data, allowing no or a maximum of one missing follow-up measurement. Furthermore, we additionally adjusted for physical activity and Mediterranean diet score, as these data were missing in more participants. Finally, we replaced office systolic blood pressure with 24 h ambulatory systolic blood pressure, waist circumference with BMI and total-to-HDL-cholesterol ratio with triacylglycerols. A two-sided *p* value <0.05 was considered statistically significant.

## Results

### General characteristics of the study population

During 10,932 person-years of follow-up, 265 (9.3%) participants developed clinically relevant depressive symptoms (PHQ-9 ≥10; average follow-up time of 2.5 ± 1.2 years), which yields an incidence rate of 24 cases per 1000 person-years. Participants not included in the analyses (*n* = 603) were statistically significantly younger, had a lower level of education, less often had a partner, had higher levels of hyperglycaemia and insulin resistance, and had a worse cardiometabolic risk profile than participants included in the analyses (data not shown).

Table 1 shows the general characteristics of the study population at baseline, stratified for incident depressive symptoms. Participants had a mean age of 59.9 ± 8.1 years and 48.8% were women. Participants with incident depressive symptoms had a worse cardiometabolic risk profile compared with participants free of depressive symptoms.

**Table 1:** General characteristics and markers of hyperglycaemia and insulin resistance according to incident depression status

Characteristic	No depressive symptoms at baseline and follow-up (*n* = 2583)	Incident depressive symptoms (PHQ-9 ≥10) (*n* = 265)	*p* value
Demographics			
Age (years)	59.9 ± 8.1	59.8 ± 8.2	0.768
Sex, *n* (% female)	1263 (48.9)	127 (47.9)	0.796
Educational level, low/medium/high, *n* (%)	766/723/1048 (30.2/28.5/41.3)	114/79/69 (43.5/30.2/26.3)	<0.001
Partner status, *n* (%) (partner)	2187 (85.8)	218 (82.9)	
Depression			
Depressive symptoms (PHQ-9 score)	2.0 ± 2.1	4.5 ± 2.8	<0.001
MDD (MINI), *n* (%)	23 (1.0)	18 (7.1)	<0.001
Anti-depressive medication, *n* (%)	124 (4.8)	40 (15.1)	<0.001
Cardiovascular risk factors			
BMI (kg/m^2^)	26.7 ± 4.3	28.5 ± 5.2	<0.001
Waist circumference (cm)	94.7 ± 13.1	100.0 ± 15.2	<0.001
Office systolic BP (mmHg)	134.6 ± 17.9	135.6 ± 19.5	0.397
Office diastolic BP (mmHg)	76.1 ± 9.7	76.6 ± 11.2	0.480
Antihypertensive medication, *n* (%)	953 (37.0)	133 (50.2)	<0.001
Hypertension, *n* (%)	1405 (54.5)	165 (62.3)	0.016
Total-to-HDL-cholesterol ratio	3.6 ± 1.1	3.9 ± 1.3	0.003
Triacylglycerols (mmol/l)	1.4 ± 0.8	1.7 ± 1.3	<0.001
Lipid-modifying medication, *n* (%)	860 (33.4)	109 (41.1)	0.012
eGFR (ml min^−1^ 1.73 m^−2^)	88.3 ± 14.3	86.6 ± 16.6	0.124
Albuminuria, normal/micro/macro, *n* (%)	2238/157/15 (92.9/6.5/0.6)	214/29/4 (86.6/11.7/1.6)	<0.001
History of CVD, *n* (%)	376 (15.0)	66 (25.4)	<0.001
Type 2 diabetes mellitus, *n* (%)	612 (23.7)	110 (41.5)	<0.001
Diabetes medication (all types), *n* (%)	456 (17.7)	92 (34.7)	<0.001
Diabetes medication (insulin), *n* (%)	102 (3.9)	32 (12.1)	<0.001
Lifestyle factors			
Smoking, never/former/current, *n* (%)	929/1340/278 (36.5/52.6/10.9)	77/129/56 (29.4/49.2/21.4)	<0.001
Alcohol use, none/low/high, *n* (%)	399/1443/702 (15.7/56.7/27.6)	68/144/49 (26.1/55.2/18.8)	<0.001
Physical activity (h/week)	14.4 ± 8.0	13.0 ± 8.8	0.008
Mediterranean diet score	4.5 ± 1.7	4.2 ± 1.6	0.008
Markers of hyperglycaemia and insulin resistance			
Fasting plasma glucose (mmol/l)	5.9 ± 1.4	6.6 ± 2.2	<0.001
2 h post-load glucose (mmol/l)	7.6 ± 4.0	8.9 ± 4.9	<0.001
HbA_1c_ (mmol/mol)	39.9 ± 8.5	44.4 ± 11.5	<0.001
HbA_1c_ (%)	5.8 ± 0.8	6.2 ± 1.1	<0.001
SAF (AU)	2.4 ± 0.5	2.5 ± 0.6	0.010
HOMA-IR	1.7 ± 1.1	1.9 ± 1.2	0.021
ISI	4.1 ± 2.7	3.6 ± 2.4	0.008

Data are presented as mean±SD or number and percentage, as appropriate

AU, arbitrary units

In cross-sectional analyses, markers of hyperglycaemia and insulin resistance were associated with prevalent depressive symptoms. However, associations with insulin resistance were attenuated after adjustment for cardiovascular and lifestyle factors, in particular waist circumference (Table 2).

**Table 2:** Cross-sectional associations of markers of hyperglycaemia and insulin resistance with prevalent depressive symptoms

Model	Prevalent depressive symptoms Rate ratio (95% CI)	*p* value	Prevalent clinically relevant depressive symptoms (PHQ-9 ≥10) OR (95% CI)	*p* value
Markers of hyperglycaemia				
Fasting plasma glucose (per 1 SD)				
Model 1	1.08 (1.04, 1.12)	<0.001	1.30 (1.15, 1.46)	<0.001
Model 2	1.15 (1.10, 1.20)	<0.001	1.41 (1.25, 1.60)	<0.001
Model 3	1.08 (1.03, 1.13)	0.001	1.17 (1.00, 1.36)	0.045
Model 4	1.07 (1.02, 1.12)	0.008	1.13 (0.97, 1.32)	0.130
2 h post-load glucose (per 1 SD)				
Model 1	1.04 (1.00, 1.08)	0.074	1.19 (1.01, 1.41)	0.042
Model 2	1.10 (1.05, 1.15)	<0.001	1.35 (1.13, 1.61)	0.001
Model 3	1.03 (0.98, 1.09)	0.298	1.06 (0.84, 1.33)	0.619
Model 4	1.02 (0.97, 1.08)	0.407	1.05 (0.84, 1.33)	0.656
HbA_1c_ (per 1 SD)				
Model 1	1.12 (1.07, 1.16)	<0.001	1.42 (1.26, 1.61)	<0.001
Model 2	1.18 (1.13, 1.23)	<0.001	1.54 (1.35, 1.75)	<0.001
Model 3	1.11 (1.06, 1.16)	<0.001	1.30 (1.11, 1.52)	0.001
Model 4	1.08 (1.03, 1.13)	0.002	1.21 (1.03, 1.42)	0.022
SAF (per 1 SD)				
Model 1	1.03 (0.99, 1.08)	0.122	1.18 (1.00, 1.39)	0.051
Model 2	1.11 (1.06, 1.16)	<0.001	1.43 (1.19, 1.72)	<0.001
Model 3	1.07 (1.02, 1.13)	0.004	1.31 (1.07, 1.60)	0.009
Model 4	1.04 (0.99, 1.09)	0.152	1.18 (0.95, 1.45)	0.129
Markers of insulin resistance				
ISI (per SD)^a^				
Model 1	1.04 (1.00, 1.09)	0.056	1.16 (0.93, 1.45)	0.182
Model 2	1.10 (1.05, 1.15)	<0.001	1.29 (1.02, 1.63)	0.033
Model 3	1.02 (0.96, 1.07)	0.598	0.89 (0.69, 1.15)	0.363
Model 4	1.01 (0.96, 1.07)	0.621	0.88 (0.68, 1.14)	0.323
HOMA-IR (per SD)				
Model 1	1.07 (1.02, 1.12)	0.004	1.20 (1.02, 1.42)	0.032
Model 2	1.12 (1.07, 1.17)	<0.001	1.28 (1.08, 1.53)	0.005
Model 3	1.03 (0.97, 1.09)	0.345	0.95 (0.75, 1.20)	0.665
Model 4	1.02 (0.96, 1.08)	0.485	0.93 (0.73, 1.18)	0.561

Total number of participants included in model 1: *n* = 3121 (fasting plasma glucose); *n* = 2920 (2 h post-load glucose); *n* = 3115 (HbA_1c_); *n* = 2959 (SAF); *n* = 2709 (ISI); and *n* = 2814 (HOMA-IR)

Number of prevalent depression cases in model 1: *n* = 138 (fasting plasma glucose); *n* = 113 (2 h post-load glucose); *n* = 139 (HbA_1c_); *n* = 131 (SAF); *n* = 101 (ISI); and HOMA-IR (*n* = 110)

Model 1: crude

Model 2: adjusted for age, sex and educational level. Data missing, *n* = 60 (fasting plasma glucose)

Model 3: additionally adjusted for waist circumference, office systolic blood pressure, antihypertensive medication, total-to-HDL-cholesterol ratio, lipid-modifying medication and history of CVD. Additional missing data, *n* = 128 (fasting plasma glucose)

Model 4: additionally adjusted for smoking behaviour and alcohol use. Additional missing data, *n* = 97 (fasting plasma glucose)

^a^The reciprocal was used for the ISI (1/ISI)

### Associations of hyperglycaemia with incident depressive symptoms

Table 3 shows the associations of markers of hyperglycaemia with incident depressive symptoms. Fasting plasma glucose, 2 h post-load glucose and HbA_1c_ levels were associated with an increased risk for incident depressive symptoms after full adjustment (HR 1.20 [95% CI 1.08, 1.33]; HR 1.25 [1.08, 1.44]; and HR 1.22 [1.09, 1.37] per SD, respectively). SAF was not associated with incident depressive symptoms (HR 0.99 [0.86, 1.13] per SD). No interactions were found with regard to sex for fasting plasma glucose (*p*-interaction = 0.981), 2 h post-load glucose (*p*-interaction = 0.234) and HbA_1c_ (*p*-interaction = 0.686). There was an interaction with sex for SAF (*p*-interaction = 0.031); however, associations were not significant in stratified analyses for men (HR 1.13 [0.94, 1.37] per SD) or women (HR 0.83 [0.67, 1.02] per SD).

**Table 3:** Associations of markers of hyperglycaemia and insulin resistance with incident depressive symptoms

Model	Incident depressive symptoms (PHQ-9 ≥10) HR (95% CI)	*p* value
Markers of hyperglycaemia		
Fasting plasma glucose (per 1 SD)		
Model 1	1.35 (1.25, 1.46)	<0.001
Model 2	1.33 (1.22, 1.45)	<0.001
Model 3	1.21 (1.09, 1.34)	<0.001
Model 4	1.20 (1.08, 1.33)	0.001
2 h post-load glucose (per 1 SD)		
Model 1	1.32 (1.18, 1.47)	<0.001
Model 2	1.29 (1.14, 1.45)	<0.001
Model 3	1.26 (1.09, 1.46)	0.002
Model 4	1.25 (1.08, 1.44)	0.003
HbA_1c_ (per 1 SD)		
Model 1	1.44 (1.32, 1.57)	<0.001
Model 2	1.40 (1.27, 1.53)	<0.001
Model 3	1.28 (1.15, 1.43)	<0.001
Model 4	1.22 (1.09, 1.37)	0.001
SAF (per 1 SD)		
Model 1	1.15 (1.02, 1.30)	0.019
Model 2	1.12 (0.99, 1.28)	0.075
Model 3	1.06 (0.93, 1.22)	0.401
Model 4	0.99 (0.86, 1.13)	0.831
Markers of insulin resistance		
ISI (per SD)^a^		
Model 1	1.22 (1.05, 1.43)	0.010
Model 2	1.21 (1.03, 1.42)	0.018
Model 3	1.03 (0.86, 1.23)	0.748
Model 4	1.03 (0.86, 1.23)	0.783
HOMA-IR (per SD)		
Model 1	1.19 (1.06, 1.34)	0.003
Model 2	1.19 (1.05, 1.34)	0.006
Model 3	0.99 (0.84, 1.17)	0.962
Model 4	0.98 (0.83, 1.15)	0.766

Total number of participants included in model 1: *n* = 2846 (fasting plasma glucose); *n* = 2683 (2 h post-load glucose); *n* = 2842 (HbA_1c_); *n* = 2710 (SAF); *n* = 2496 (ISI); and *n* = 2585 (HOMA-IR)

Number of incident depression cases in model 1: *n* = 265 (fasting plasma glucose); *n* = 226 (2 h post-load glucose); *n* = 264 (HbA_1c_); *n* = 254 (SAF); *n* = 212 (ISI); and *n* = 220 (HOMA-IR)

Model 1: crude

Model 2: adjusted for age, sex and educational level. Data missing, *n* = 49 (fasting plasma glucose)

Model 3: additionally adjusted for waist circumference, office systolic blood pressure, antihypertensive medication, total-to-HDL-cholesterol ratio, lipid-modifying medication and history of CVD. Additional missing data, *n* = 71 (fasting plasma glucose)

Model 4: additionally adjusted for smoking behaviour and alcohol use. Additional missing data, *n* = 133 (fasting plasma glucose)

^a^The reciprocal was used for the ISI to define it as risk factor (1/ISI)

### Associations of insulin resistance with incident depressive symptoms

Table 3 shows the associations of insulin resistance with incident depressive symptoms. A lower ISI and a higher HOMA-IR were associated with an increased risk for incident depressive symptoms after adjustment for age, sex and educational level (HR 1.21 [1.03, 1.42] and HR 1.19 [1.05, 1.34] per SD, respectively). After additional adjustment for cardiovascular risk factors, these associations were attenuated (HR 1.03 [0.86, 1.23] and HR 0.99 [0.84, 1.17] per SD, respectively). These attenuations were mainly caused by waist circumference (model 2 additionally adjusted for waist circumference: HR 1.04 [0.87, 1.24] and HR 1.02 [0.87, 1.19] per SD, respectively). No interaction with regard to sex was found for ISI (*p*-interaction = 0.589) and HOMA-IR (*p*-interaction = 0.621).

### Additional analyses

Results of additional analyses are shown in Table 4. Additional adjustment for type 2 diabetes, and excluding participants with type 2 diabetes from the analyses, did not materially change the associations. As expected, additional adjustment for type 2 diabetes attenuated the associations, but HRs remained directionally similar.

**Table 4:** Additional analyses for associations of markers of hyperglycaemia with incident depressive symptoms

Model	Incident clinically relevant depressive symptoms (PHQ-9 ≥10) HR (95% CI)	*p* value
Fasting plasma glucose (per 1 SD)		
Model 4	1.20 (1.08, 1.33)	0.001
Model 5: model 4 + type 2 diabetes	1.12 (0.99, 1.27)	0.085
Model 6: model 4 excl. type 2 diabetes (excluded data *n* = 683)	1.35 (0.81, 2.25)	0.255
Model 7: model 4 + antidepressant medication	1.20 (1.08, 1.33)	0.001
Model 8: model 4 excl. antidepressant users (missing data *n* = 152)	1.18 (1.05, 1.33)	0.006
Model 9: model 4 excl. baseline MDD (excluded data *n* = 150)	1.19 (1.06, 1.33)	0.003
Model 10: model 4 excl. lifetime MDD (excluded data *n* = 897)	1.06 (0.87, 1.29)	0.580
Model 11: model 4 + physical activity (missing data *n* = 160)	1.19 (1.07, 1.33)	0.002
Model 12: model 4 + Mediterranean diet (missing data *n* = 123)	1.18 (1.06, 1.32)	0.002
Model 13: model 4 replacing office SBP for 24 h SBP (missing data *n* = 294)	1.19 (1.06, 1.33)	0.003
Model 14: model 4 replacing waist circumference for BMI	1.21 (1.09, 1.34)	<0.001
Model 15: model 4 replacing total-to-HDL-cholesterol ratio for triacylglycerols	1.17 (1.05, 1.30)	0.004
2 h post-load glucose (per 1 SD)		
Model 4	1.25 (1.08, 1.44)	0.003
Model 5: model 4 + type 2 diabetes	1.16 (0.93, 1.45)	0.192
Model 6: model 4 excl. type 2 diabetes (excluded data *n* = 539)	1.19 (0.78, 1.83)	0.419
Model 7: model 4 + antidepressant medication	1.27 (1.10, 1.47)	0.001
Model 8: model 4 excl. antidepressant users (missing data *n* = 132)	1.26 (1.08, 1.47)	0.003
Model 9: model 4 excl. baseline MDD (excluded data *n* = 143)	1.23 (1.05, 1.44)	0.009
Model 10: model 4 excl. lifetime MDD (excluded data *n* = 840)	1.18 (0.94, 1.49)	0.163
Model 11: model 4 + physical activity (missing data *n* = 153)	1.22 (1.04, 1.42)	0.014
Model 12: model 4 + Mediterranean diet (missing data *n* = 116)	1.24 (1.06, 1.44)	0.006
Model 13: model 4 replacing office SBP for 24 h SBP (missing data *n* = 274)	1.23 (1.05, 1.44)	0.008
Model 14: model 4 replacing waist circumference for BMI	1.25 (1.08, 1.44)	0.002
Model 15: model 4 replacing total-to-HDL-cholesterol ratio for triacylglycerols	1.21 (1.04, 1.40)	0.015
HbA_1c_ (per 1 SD)		
Model 4	1.22 (1.09, 1.37)	0.001
Model 5: model 4 + type-2 diabetes	1.14 (1.00, 1.31)	0.057
Model 6: model 4 excl. type-2 diabetes (excluded data *n* = 684)	1.23 (0.82, 1.83)	0.318
Model 7: model 4 + antidepressant medication	1.23 (1.10, 1.38)	<0.001
Model 8: model 4 excl. antidepressant users (missing data *n* = 152)	1.18 (1.03, 1.34)	0.017
Model 9: model 4 excl. baseline MDD (excluded data *n* = 150)	1.21 (1.07, 1.37)	0.003
Model 10: model 4 excl. lifetime MDD (excluded data *n* = 894)	1.08 (0.87, 1.33)	0.486
Model 11: model 4 + physical activity (missing data *n* = 160)	1.25 (1.11, 1.41)	<0.001
Model 12: model 4 + Mediterranean diet (missing data *n* = 123)	1.20 (1.06, 1.35)	0.004
Model 13: model 4 replacing office SBP for 24 h SBP (missing data *n* = 294)	1.23 (1.08, 1.41)	0.002
Model 14: model 4 replacing waist circumference for BMI	1.23 (1.10, 1.38)	<0.001
Model 15: model 4 replacing total-to-HDL-cholesterol ratio for triacylglycerols	1.20 (1.07, 1.35)	0.002
SAF (per 1 SD)		
Model 4	0.99 (0.86, 1.13)	0.831
Model 5: model 4 + type-2 diabetes	0.94 (0.85, 1.11)	0.606
Model 6: model 4 excl. type-2 diabetes (excluded data *n* = 658)	0.87 (0.72, 1.05)	0.154
Model 7: model 4 + antidepressant medication	1.00 (0.87, 1.15)	0.975
Model 8: model 4 excl. antidepressant users (missing data *n* = 147)	1.01 (0.87, 1.18)	0.852
Model 9: model 4 excl. baseline MDD (excluded data *n* = 145)	1.01 (0.87, 1.17)	0.876
Model 10: model 4 excl. lifetime MDD (excluded data *n* = 854)	1.00 (0.81, 1.25)	0.985
Model 11: model 4 + physical activity (missing data *n* = 150)	1.03 (0.89, 1.19)	0.696
Model 12: model 4 + Mediterranean diet (missing data *n* = 117)	1.00 (0.87, 1.16)	0.986
Model 13: model 4 replacing office SBP for 24 h SBP (missing data *n* = 280)	0.93 (0.81, 1.08)	0.353
Model 14: model 4 replacing waist circumference for BMI	0.99 (0.86, 1.14)	0.897
Model 15: model 4 replacing total-to-HDL-cholesterol ratio for triacylglycerols	0.98 (0.86, 1.13)	0.800

Total number of participants in model 4: *n* = 2714 (fasting plasma glucose); *n* = 2565 (2 h post-load glucose); *n* = 2710 (HbA_1c_); *n* = 2582 (SAF)

Incident depressive symptoms in model 4: *n* = 254 (fasting plasma glucose); *n* = 217 (2 h post-load glucose); *n* = 253 (HbA_1c_); and *n* = 244 (SAF)

Model 4 is adjusted for age, sex, educational level, waist circumference, office systolic blood pressure, antihypertensive medication, total-to-HDL-cholesterol ratio, lipid-modifying medication, history of CVD, smoking behaviour and alcohol use

Excl., excluding; SBP, systolic blood pressure

Adjustments to reduce potential misclassification of participants with subthreshold depression did not materially change our results. Furthermore, applying stricter rules on the follow-up data, allowing no or a maximum of one missing follow-up measurement for the control participants, did not materially change our results (data not shown).

Similar strengths of the associations were found after additional adjustment for physical activity or Mediterranean diet score. Furthermore, our results were not materially changed by replacing office systolic blood pressure with 24 h ambulatory systolic blood pressure, replacing waist circumference with BMI or replacing total-to-HDL-cholesterol ratio with triacylglycerols.

## Discussion

This population-based study demonstrates that fasting plasma glucose, 2 h post-load glucose and HbA_1c_ were associated with incident depressive symptoms, with an increased risk of ~20% per SD higher level of hyperglycaemia markers. These associations were independent of demographical, cardiovascular and lifestyle-related risk factors, and were similar in women and men. The association of insulin resistance with incident depressive symptoms was explained by cardiovascular risk factors (waist circumference). Our results suggest that hyperglycaemia precedes the development of depression, and may be directly involved in its aetiology.

Our finding that hyperglycaemia is associated with incident depressive symptoms corroborates and further extends previous evidence of an association between type 2 diabetes and incident depression [30], and provides additional evidence that hyperglycaemia as such may be involved in the development of depression. This is in line with results of a large-scale cross-sectional study that showed an association between both diagnosed and undiagnosed diabetes and higher prevalence of depression [31]. Although a previous meta-analysis concluded that hyperglycaemia is unlikely to be causally related to incident depressive symptoms [8], this study did not investigate a linear contribution of hyperglycaemia to the incidence of depression.

Several pathophysiological pathways may explain the association between hyperglycaemia and incident depression. Hyperglycaemia is associated with generalised microvascular dysfunction [32], which may consequently lead to cerebral small vessel disease and subsequent depression [33]. Indeed, a recent meta-analysis showed that cerebrovascular damage was associated with incident depression [34]. Optimising blood glucose levels is the most effective therapy to prevent the development of microvascular complications in type 2 diabetes, and could potentially also contribute to preventing or slowing down the development of depressive symptoms. Alternatively, suboptimal blood glucose levels may also identify those individuals at high risk for depression. Furthermore, hyperglycaemia has been associated with low-grade inflammation [35], which in turn has been associated with cerebrovascular damage [36] and incident depression as well [37]. In support of this potential mechanism, several studies have shown that treatment resistance to antidepressants is associated with low-grade inflammation [38] and that anti-inflammatory therapy may be beneficial to individuals with depression [39]. Moreover, hyperglycaemia may activate the polyol pathway which induces oxidative stress, increases lipid peroxidation and imbalances the generation of ROS [40]. These processes may lead to apoptosis in the brain, which may eventually lead to depression via shrinkage of specific brain structures (atrophy) [41]. This assumption is supported by a stronger association between oxidative stress and depression in individuals with IGM and type 2 diabetes than in those with NGM [42]. Furthermore, previous studies have assumed that diabetes may increase risk of depression because of disease burden [8]. However, disease burden alone may be not sufficient to explain the association between hyperglycaemia and incident depression, since 65% of the association remained after additional adjustment for type 2 diabetes. In addition, the suggestion that somatic symptoms may explain this association is unlikely, as a previous study of our group has shown that affective and somatic symptoms do not differ between individuals with and individual without type 2 diabetes [43].

We found no association between SAF and incident depressive symptoms, although earlier cross-sectional analyses in a smaller dataset (*n* = 866) from The Maastricht Study did show an association between higher SAF and prevalent depression [20]. SAF is thought to represent the accumulation of fluorescent AGEs in the skin, but may be a less specific measure of hyperglycaemia, as it also measures other fluorescent proteins in the skin and does not reflect non-fluorescent AGEs [44]. Nevertheless, there are currently no other prospective studies available that have assessed this association. Therefore, this finding warrants replication in other prospective population-based studies in order to draw firm conclusions.

We found that the association of insulin resistance with incident depression was explained by CVD risk factors, in particular central obesity. This is in contrast with results of the Whitehall II Study, the Caerphilly Study and the Pittsburgh Healthy Heart Project, which did not show an association between insulin resistance and incident depression after adjustment for age only [15, 16]. Furthermore, our results contrast with the results of the Health in Men Study, which did show an association between higher insulin resistance and incident depression after adjustment for cardiovascular risk factors including central obesity [14]. However, the Health in Men Study only included older men aged 70–93 years, which hinders direct comparison with our somewhat younger population. There are several explanations for the attenuation of the association between insulin resistance and incident depressive symptoms after adjustment for central obesity. First, central obesity may be on the causal pathway from insulin resistance to depression, which might have resulted in overadjustment. Second, as performing clamps is not feasible in large-scale studies, we used surrogate markers of insulin resistance. These markers moderately reflect hepatic and muscular insulin resistance, which may or may not coincide with cerebral insulin resistance [45]. Consequently, we cannot fully exclude the possibility that cerebral insulin resistance is involved in the development of depression. Third, insulin resistance is less precisely measured than hyperglycaemia. The use of surrogate markers of insulin resistance may have created more noise in the data as compared with the direct markers of hyperglycaemia. Alternatively, hyperglycaemia may be one of the mechanisms linking insulin resistance to depression. Obesity is associated with the development of insulin resistance, but only individuals who lack sufficient insulin secretion to match the degree of insulin resistance will develop type 2 diabetes [46].

The association of hyperglycaemia with an increased risk of depressive symptoms has important clinical implications. First, professionals in diabetes care should be aware of the prevalence of depression, and use diagnostic skills to recognise and treat depression properly. For this, specific guidelines to identify and manage depressive symptoms in diabetes care have been developed [47]. In addition to these guidelines, it is important to distinguish between need for treatment and a high score on a questionnaire [48]. Since depression in individuals with type 2 diabetes is often persistent [6], and is related to suboptimal blood glucose levels [3], early recognition and treatment of depressive symptoms could have a favourable effect on the outcome of both diseases [49]. Considering the high comorbidity of depression and type 2 diabetes, integrated care approaches that treat these conditions jointly need to be implemented in diabetes care.

Strengths of our study include its large sample size and population-based longitudinal design; the oversampling of individuals with type 2 diabetes which results in more variability within the high ranges of hyperglycaemia; the annual assessment of the PHQ-9 to assess depressive symptoms over a 4 year period; the comparable incidence rate of depression to other population-based studies; the use of multiple continuous markers of hyperglycaemia; the extensive assessment of potential confounders; and the execution of several sensitivity analyses.

This study has some limitations. First, there could have been selection and/or attrition bias, which is inherent to prospective population-based studies; individuals with more severe depressive symptoms or with greater comorbidity may have been more likely not to participate or to withdraw, which may have led to an underestimation of the observed associations. Second, the study population was relatively well treated with regard to glucose metabolism, which may mean that the effects of fasting plasma glucose, 2 h post-load glucose and HbA_1c_ on incident depression were suppressed. Estimates of post-load glucose, ISI and HOMA-IR, did not include insulin users, which may have led to an underestimation of the observed findings in more severe type 2 diabetes. Third, the population was mainly of white ethnicity and aged 40–75 years, which should be considered when extrapolating these findings to other populations. Fourth, we measured depressive symptoms with the PHQ-9 questionnaire. High scores on this questionnaire are suggestive for depressive symptoms, but do not necessarily equate with MDD. Finally, because follow-up data were only available for depression data, we could not rule reverse causality; there might be a reciprocal relation in which depression may also lead to hyperglycaemia.

## Conclusion

In conclusion, we showed that higher levels of hyperglycaemia were associated with incident depressive symptoms in a population-based setting, independent of major demographical, cardiovascular and lifestyle risk factors. The association of insulin resistance with incident depressive symptoms was dependent on cardiovascular risk factors, in particular, central obesity. These findings establish a temporal relation between hyperglycaemia and incident depressive symptoms, supporting the concept that hyperglycaemia itself is involved in the aetiology of depression, and thus may provide a potential target for the prevention of depression in individuals with and without type 2 diabetes.

## Electronic supplementary material

ESM Methods (PDF 113 kb)

## Data Availability

The data of this study derive from The Maastricht Study, but restrictions apply to the availability of these data, which were used under license for the current study. Data are, however, available from the authors upon reasonable request and with permission of The Maastricht Study management team.

## Abbreviations

IGM: Impaired glucose metabolism
ISI: Insulin sensitivity index
MDD: Major depressive disorder
NGM: Normal glucose metabolism
PHQ-9: Nine-item Patient Health Questionnaire
ROS: Reactive oxygen species
SAF: Skin autofluorescence

## References

[1] Roy T, Lloyd CE (2012). Epidemiology of depression and diabetes: a systematic review. J Affect Disord.

[2] Schram MT, Baan CA, Pouwer F (2009). Depression and quality of life in patients with diabetes: a systematic review from the European Depression in Diabetes (EDID) Research Consortium. Curr Diabetes Rev.

[3] Pouwer F, Nefs G, Nouwen A (2013). Adverse effects of depression on glycemic control and health outcomes in people with diabetes: a review. Endocrinol Metab Clin N Am.

[4] Katon W, Lyles CR, Parker MM, Karter AJ, Huang ES, Whitmer RA (2012). Association of depression with increased risk of dementia in patients with type 2 diabetes: the Diabetes and Aging Study. Arch Gen Psychiatry.

[5] Molosankwe I, Patel A, Gagliardino JJ, Knapp M, McDaid D (2012). Economic aspects of the association between diabetes and depression: a systematic review. J Affect Disord.

[6] Nefs G, Pouwer F, Denollet J, Pop V (2012). The course of depressive symptoms in primary care patients with type 2 diabetes: results from the Diabetes, Depression, Type D Personality Zuidoost-Brabant (DiaDDZoB) Study. Diabetologia.

[7] van Sloten T, Schram M (2018). Understanding depression in type 2 diabetes: a biological approach in observational studies. F1000Res.

[8] Tong A, Wang X, Li F, Xu F, Li Q, Zhang F (2016). Risk of depressive symptoms associated with impaired glucose metabolism, newly diagnosed diabetes, and previously diagnosed diabetes: a meta-analysis of prospective cohort studies. Acta Diabetol.

[9] Pieper L, Dirmaier J, Klotsche J (2011). Longitudinale Assoziationen zwischen depressiven Symptomen und Typ-2-Diabetes sowie deren Auswirkung auf die Mortalität von Hausarztpatienten. Bundesgesundheitsbl Gesundheitsforsch Gesundheitsschutz.

[10] Icks A, Albers B, Haastert B (2013). Risk for high depressive symptoms in diagnosed and previously undetected diabetes: 5-year follow-up results of the Heinz Nixdorf Recall Study. PLoS One.

[11] Okumiya K, Fujisawa M, Sakamoto R (2015). Effect of early diagnosis and lifestyle modification on depressive symptoms in community-dwelling elderly adults with glucose intolerance: 5-year longitudinal study. J Am Geriatr Soc.

[12] Demakakos P, Zaninotto P, Nouwen A (2014). Is the association between depressive symptoms and glucose metabolism bidirectional? Evidence from the English Longitudinal Study of Ageing. Psychosom Med.

[13] Golden SH, Lazo M, Carnethon M (2008). Examining a bidirectional association between depressive symptoms and diabetes. JAMA.

[14] Ford AH, Flicker L, Hankey GJ (2015). Insulin resistance and depressive symptoms in older men: the Health in Men Study. Am J Geriatr Psychiatry.

[15] Lawlor DA, Ben-Shlomo Y, Ebrahim S (2005). Insulin resistance and depressive symptoms in middle aged men: findings from the Caerphilly prospective cohort study. BMJ.

[16] Akbaraly TN, Kumari M, Head J (2013). Glycemia, insulin resistance, insulin secretion, and risk of depressive symptoms in middle age. Diabetes Care.

[17] Khambaty T, Stewart JC, Muldoon MF, Kamarck TW (2014). Depressive symptom clusters as predictors of 6-year increases in insulin resistance: data from the Pittsburgh Healthy Heart Project. Psychosom Med.

[18] Schram MT, Sep SJ, van der Kallen CJ (2014). The Maastricht Study: an extensive phenotyping study on determinants of type 2 diabetes, its complications and its comorbidities. Eur J Epidemiol.

[19] World Health Organization (2006). Definition and diagnosis of diabetes mellitus and intermediate hyperglycaemia: report of a WHO/IDF consultation.

[20] van Dooren FE, Pouwer F, Schalkwijk CG (2017). Advanced glycation end product (AGE) accumulation in the skin is associated with depression: the Maastricht Study. Depress Anxiety.

[21] Singh B, Saxena A (2010). Surrogate markers of insulin resistance: a review. World J Diabetes.

[22] DeFronzo RA, Matsuda M (2010). Reduced time points to calculate the composite index. Diabetes Care.

[23] Matsuda M, DeFronzo RA (1999). Insulin sensitivity indices obtained from oral glucose tolerance testing: comparison with the euglycemic insulin clamp. Diabetes Care.

[24] The Oxford Centre for Diabetes, Endocrinology and Metabolism Diabetes Trials Unit. HOMA2 Calculator. Available from https://www.dtu.ox.ac.uk/homacalculator/. Accessed 7 Feb 2019

[25] Wallace TM, Levy JC, Matthews DR (2004). Use and abuse of HOMA modeling. Diabetes Care.

[26] Kroenke K, Spitzer R, Williams J (2001). The PHQ-9: validity of a brief depression severity measure. Gen Intern Med.

[27] Bell CC (1994). DSM-IV: diagnostic and statistical manual of mental disorders. JAMA.

[28] Pettersson A, Bostrom KB, Gustavsson P, Ekselius L (2015). Which instruments to support diagnosis of depression have sufficient accuracy? A systematic review. Nord J Psychiatry.

[29] Sheehan DV, Lecrubier Y, Sheehan KH (1998). The Mini-International Neuropsychiatric Interview (MINI): the development and validation of a structured diagnostic psychiatric interview for DSM-IV and ICD-10. J Clin Psychiatry.

[30] Rotella F, Mannucci E (2013). Diabetes mellitus as a risk factor for depression. A meta-analysis of longitudinal studies. Diabetes Res Clin Pract.

[31] Meurs M, Roest AM, Wolffenbuttel BH, Stolk RP, de Jonge P, Rosmalen JG (2016). Association of depressive and anxiety disorders with diagnosed versus undiagnosed diabetes: an epidemiological study of 90,686 participants. Psychosom Med.

[32] Stehouwer CD (2018). Microvascular dysfunction and hyperglycemia: a vicious cycle with widespread consequences. Diabetes.

[33] Wardlaw JM, Smith EE, Biessels GJ (2013). Neuroimaging standards for research into small vessel disease and its contribution to ageing and neurodegeneration. Lancet Neurol.

[34] van Agtmaal MJ, Houben AJ, Pouwer F, Stehouwer CD, Schram MT (2017). Association of microvascular dysfunction with late-life depression: a systematic review and meta-analysis. JAMA Psychiatry.

[35] Wang X, Bao W, Liu J (2013). Inflammatory markers and risk of type 2 diabetes: a systematic review and meta-analysis. Diabetes Care.

[36] Leonard BE (2007). Inflammation, depression and dementia: are they connected?. Neurochem Res.

[37] Pasco JA, Nicholson GC, Williams LJ (2010). Association of high-sensitivity C-reactive protein with de novo major depression. Br J Psychiatry.

[38] Strawbridge R, Arnone D, Danese A, Papadopoulos A, Herane Vives A, Cleare AJ (2015). Inflammation and clinical response to treatment in depression: a meta-analysis. Eur Neuropsychopharmacol.

[39] Kappelmann N, Lewis G, Dantzer R, Jones PB, Khandaker GM (2018). Antidepressant activity of anti-cytokine treatment: a systematic review and meta-analysis of clinical trials of chronic inflammatory conditions. Mol Psychiatry.

[40] Sima AA, Kamiya H, Li ZG (2004). Insulin, C-peptide, hyperglycemia, and central nervous system complications in diabetes. Eur J Pharmacol.

[41] McKernan DP, Dinan TG, Cryan JF (2009). “Killing the Blues”: a role for cellular suicide (apoptosis) in depression and the antidepressant response?. Prog Neurobiol.

[42] Van Sloten T, Schram MT, Adriaanse M (2014). Endothelial dysfunction is associated with a greater depressive symptom score in a general elderly population: the Hoorn Study. Psychol Med.

[43] Janssen EP, Köhler S, Stehouwer CD et al (2016) The Patient Health Questionnaire-9 as a screening tool for depression in individuals with type 2 diabetes mellitus: the Maastricht Study. J Am Geriatr Soc 64(11). 10.1111/jgs.14388 10.1111/jgs.14388 27783384

[44] Meerwaldt R, Graaff R, Oomen P (2004). Simple non-invasive assessment of advanced glycation endproduct accumulation. Diabetologia.

[45] Banks WA, Owen JB, Erickson MA (2012). Insulin in the brain: there and back again. Pharmacol Ther.

[46] Al-Goblan AS, Al-Alfi MA, Khan MZ (2014) Mechanism linking diabetes mellitus and obesity. Diabetes Metab Syndr Obes 7:587–591 10.2147/DMSO.S67400 PMC4259868 25506234

[47] Young-Hyman D, De Groot M, Hill-Briggs F, Gonzalez JS, Hood K, Peyrot M (2016). Psychosocial care for people with diabetes: a position statement of the American Diabetes Association. Diabetes Care.

[48] Snoek FJ, Hermanns N, de Wit M (2018). Comment on Young-Hyman et al. Psychosocial care for people with diabetes: a position statement of the American Diabetes Association. Diabetes Care 2016;39:2126–2140. Diabetes Care.

[49] Schmitt A, Reimer A, Ehrmann D, Kulzer B, Haak T, Hermanns N (2017). Reduction of depressive symptoms predicts improved glycaemic control: secondary results from the DIAMOS study. J Diabetes Complicat.

