# Supplementary material for: The association of hyperglycaemia and insulin resistance with incident depressive symptoms over 4 years of follow-up: The Maastricht Study
Source: Diabetologia. 2020 Aug 5;63(11):2315–28. doi: 10.1007/s00125-020-05247-9 (PMC7527373; doi:10.1007/s00125-020-05247-9)
Supplement: Supplementary file 1 — (PDF 113 kb) [file 125_2020_5247_MOESM1_ESM.pdf]

Supplementary material

The Association of Hyperglycaemia and Insulin Resistance with Incident Depressive Symptoms over 4 Years of Follow-up: The Maastricht Study

Anouk FJ Geraets[1,2,3,4], Sebastian Köhler[1,3], Rutendo Muzambi[5], Casper G Schalkwijk[2,4], Anke Oenema[6,7,8], Simone JPM Eussen[7,9], Pieter C Dagnelie[2,4], Coen DA Stehouwer[2,4], Nicolaas C Schaper[2,4], Ronald MA Henry[2,4,10], Carla JH van der Kallen[2,4], Anke Wesselius[8,11], Annemarie Koster[7,12], Frans RJ Verhey[1,3] & Miranda T Schram[1,2,3,4,10]

[1]Department of Psychiatry and Neuropsychology, Maastricht University Medical Center (MUMC+), Maastricht, the Netherlands
[2]Department of Internal Medicine, Maastricht University Medical Center (MUMC+), Maastricht, the Netherlands
[3]School of Mental Health and Neuroscience (MHeNs), Faculty of Health, Medicine & Life Sciences, Maastricht University, Maastricht, the Netherlands
[4]School for Cardiovascular Diseases (CARIM), Faculty of Health, Medicine & Life Sciences, Maastricht University, Maastricht, the Netherlands
[5]Faculty of Epidemiology & Population Health, London School of Hygiene & Tropical Medicine, London, UK
[6]Department of Health Promotion, Maastricht University Medical Center (MUMC+), Maastricht, the Netherlands
[7]Care and Public Health Research Institute (CAPHRI), Faculty of Health, Medicine & Life Sciences, Maastricht University, Maastricht, the Netherlands
[8]School of Nutrition and Translational Research in Metabolism (NUTRIM), Faculty of Health, Medicine & Life Sciences, Maastricht University, Maastricht, the Netherlands
[9]Department of Epidemiology, Maastricht University Medical Center (MUMC+), Maastricht, the Netherlands
[10]Heart and Vascular Center, Maastricht University Medical Center (MUMC+), Maastricht, the Netherlands
[11]Department of Genetics & Cell Biology, Complex Genetics, Maastricht University Medical Center (MUMC+), Maastricht, the Netherlands
[12]Department of Social Medicine, Maastricht University Medical Center (MUMC+), Maastricht, the Netherlands

Methods

Measurements of cardiovascular risk factors

History of cardiovascular disease was assessed with a modified version of the Rose Questionnaire for the diagnosis of ischemic heart pain and intermittent claudication[1] and defined as self-reported myocardial infarction, and/or cerebrovascular infarction or hemorrhage, and/or percutaneous artery angioplasty of, or vascular surgery on, the coronary arteries, abdominal arteries, peripheral arteries or carotid arteries. Waist circumference was measured with a flexible plastic tape measure (Seca, Hamburg, Germany) midway between the lower rib margin and the iliac crest at the end of expiration. Weight and height were measured without shoes and wearing light clothing using a scale and stadiometer to the nearest 0.5 kg or 0.1 cm (Seca, Hamburg, Germany), and calculated BMI as weight (kg) divided by height (m^2). Venous fasting and 2 h post-load plasma glucose levels were measured by the enzymatic hexokinase method on two automatic analyzers, the Beckman Synchron LX20 (Beckman Coulter, CA, USA) for samples obtained between November 2010 and April 2012, and the Roche Cobas 6000 (Roche Diagnostics, Mannheim, Germany) for samples obtained thereafter. HbA1c was determined by ion-exchange high performance liquid chromatography. Serum concentrations of total cholesterol, HDL cholesterol, and triacylglycerols were measured by use of an automatic analyzer (Beckman Synchron LX20, Beckman Coulter Inc. Brea, USA). Office blood pressure was calculated as the mean of at least three blood pressure readings (Omron 705IT, Japan) performed after a minimum of 10 min rest. We measured serum creatinine and cystatin C[2], and 24 h urinary albumin excretion (twice) as described previously[3]. Estimated glomerular filtration rate (eGFR; ml min−1 1.73 m−2) was calculated with the Chronic Kidney Disease Epidemiology Collaboration (CKD-EPI) equation based on both serum creatinine and serum cystatin C.

Measurements of covariates

Educational level was assessed by web-based questionnaire and divided into three categories: low (none, primary or lower vocational education only), intermediate (intermediate general secondary, intermediate vocational or higher general secondary education), and high (higher vocational education or university level of education)[4]. Alcohol consumption is assessed as part of the food frequency questionnaire[5] and categorized into three categories: 1) nonconsumers, 2) low consumers (less than or equal to seven glasses per week for women and ≤ 14 glasses per week for men), and 3) high consumers (more than seven glasses per week for women and >14 glasses per week for men)[6]. All participants received an extensive web-based community healthy activities model program for seniors (CHAMPS) physical activity questionnaire[3, 7]. Activities included walking, cycling, gardening, household work, jogging/running, swimming, tennis, team sport, light and intensive exercise. The total number of hours of physical activity in the past week was used to calculate a sum score of total physical activity.

References

[1] Lend G, Fowkes F (1992) The Edinburgh Claudication Questionnaire: an improved version of the WHO/Rose Questionnaire for use in epidemiological surveys. J Clin Epidemiol 45(10): 1101-1109

[2] Martens RJ, Henry RM, Houben AJ, et al (2016) Capillary rarefaction associates with albuminuria: The Maastricht Study. J Am Soc Nephrol 27(12): 3748-3757. 10.1681/asn.2015111219

[3] Schram MT, Sep SJ, van der Kallen CJ, et al (2014) The Maastricht Study: an extensive phenotyping study on determinants of type 2 diabetes, its complications and its comorbidities. Eur J Epidemiol 29(6): 439-451. 10.1007/s10654-014-9889-0

[4] Kersten P, Cardol M, George S, Ward C, Sibley A, White B (2007) Validity of the impact on participation and autonomy questionnaire: A comparison between two countries. Disabil Rehabil 29(19): 1502-1509. 10.1080/09638280601030066

[5] Molag ML, de Vries JH, Duif N, et al (2010) Selecting informative food items for compiling food-frequency questionnaires: comparison of procedures. Br J Nutr 104(3): 446-456. 10.1017/s0007114510000401

[6] van Dooren FE, Pouwer F, Schalkwijk CG, et al (2017) Advanced glycation end product (AGE) accumulation in the skin is associated with depression: The Maastricht Study. Depress Anxiety 34(1): 59-67. 10.1002/da.22527

[7] Harada ND, Chiu V, King AC, Stewart AL (2001) An evaluation of three self-report physical activity instruments for older adults. Med Sci Sports Exerc 33(6): 962-970. 10.1097/00005768-200106000-00016
